# Towards Secure Fitness Framework Based on IoT-Enabled Blockchain Network Integrated with Machine Learning Algorithms

**DOI:** 10.3390/s21051640

**Published:** 2021-02-26

**Authors:** Faisal Jamil, Hyun Kook Kahng, Suyeon Kim, Do-Hyeun Kim

**Affiliations:** 1 Department of Computer Engineering, Jeju National University, Jejusi 63243, Korea; faisal@jejunu.ac.kr; 2 Department of Electrical and Information Engineering, Korea University, Sejong 30019, Korea; kahng@korea.ac.kr; 3 Department of Industry Cooperation, Keimyung University, Deagu 42601, Korea; sykim388@gmail.com

**Keywords:** healthcare, blockchain, fitness service, smart contract, internet of things

## Abstract

Blockchain technology has recently inspired remarkable attention due to its unique features, such as privacy, accountability, immutability, and anonymity, to name of the few. In contrast, core functionalities of most Internet of Things (IoT) resources make them vulnerable to security threats. The IoT devices, such as smartphones and tablets, have limited capacity in terms of network, computing, and storage, which make them easier for vulnerable threats. Furthermore, a massive amount of data produced by the IoT devices, which is still an open challenge for the existing platforms to process, analyze, and unearth underlying patterns to provide convenience environment. Therefore, a new solution is required to ensure data accountability, improve data privacy and accessibility, and extract hidden patterns and useful knowledge to provide adequate services. In this paper, we present a secure fitness framework that is based on an IoT-enabled blockchain network integrated with machine learning approaches. The proposed framework consists of two modules: a blockchain-based IoT network to provide security and integrity to sensing data as well as an enhanced smart contract enabled relationship and inference engine to discover hidden insights and useful knowledge from IoT and user device network data. The enhanced smart contract aims to support users with a practical application that provides real-time monitoring, control, easy access, and immutable logs of multiple devices that are deployed in several domains. The inference engine module aims to unearth underlying patterns and useful knowledge from IoT environment data, which helps in effective decision making to provide convenient services. For experimental analysis, we implement an intelligent fitness service that is based on an enhanced smart contract enabled relationship and inference engine as a case study where several IoT fitness devices are used to securely acquire user personalized fitness data. Furthermore, a real-time inference engine investigates user personalized data to discover useful knowledge and hidden insights. Based on inference engine knowledge, a recommendation model is developed to recommend a daily and monthly diet, as well as a workout plan for better and improved body shape. The recommendation model aims to facilitate a trainer formulating effective future decisions of trainee’s health in terms of a diet and workout plan. Lastly, for performance analysis, we have used Hyperledger Caliper to access the system performance in terms of latency, throughput, resource utilization, and varying orderer and peers nodes. The analysis results imply that the design architecture is applicable for resource-constrained IoT blockchain platform and it is extensible for different IoT scenarios.

## 1. Introduction

Attaining health facilities is one of the foremost fundamental rights of every human. Nowadays, many chronic diseases have arisen due to laziness and unhygienic food, which are the primary source of disability, death, and poor health. Chronic diseases are mostly driven by personal lifestyle, and different linking factors, e.g., sustained stress, inactivity, and diet smoking, which are the main factors of severe illness and death [1]. According to the study, approximately 70% of the adults’ generation spend their time sitting and working, which generates a negative healthcare outcome. On the contrary, spending 30 min. from the daily work routine on light activity reduces the risk of death and illness by 14%. The growing pervasiveness of unhealthy lifestyle and low diet implies that, one out of three adults, and one out of six children, are determined to have obesity [2,3]. It has been observed that impropoer diet impacts health, thus increasing the risk of diseases, i.e., diabetes type-2, heart stroke, and other health-related diseases. Moreover, life stress is also associated with high mental disorder and poor physical health [4,5,6].

The blockchain is based on distributed ledger technology that has been developed to store financial records that cannot be altered or manipulated. Each transaction in a blockchain is secured due to the signed transaction by the valid participant. The block in the blockchain contains a set of transactions that is digitally signed and encrypted along with the time stamp. Finally, the chain is secured with advance cryptography algorithms that make the data protected and secured. Blockchain is a distributed ledger database that comprises of six layers, i.e., the data layer, network layer, consensus mechanism, smart contract layer, service layer, and application layer. The data acquisition, authorization, and control are usually done in data layer and network layer [7]. The consensus mechanism and smart contract layer contain the functionality of a smart contract, incentive structure, and consensus protocols. Furthermore, the consensus protocol in blockchain consumes significant computational power as well as a large number of resources, which ultimately degrade the performance of in terms of throughput and latency. The service layer provides the back-end functionalities of blockchain network, which is further exposed to the application layer. The application layer provides the interaction between the back-end and the front-end. During the past several years many new technologies have been introduced in the area of blockchain, such as consensus algorithms, permission and privacy mechanisms, and smart contract. The blockchain comprises of public and private blockchain. In a private blockchain, the registered users administer and control the network. Similarly, in permissioned blockchain, the registered participant can participate in the mechanism of block creation, whereas, in permissionless blockchain, anyone can participate in the consensus and block creation mechanism. Therefore, the permissioned blockchain is transparent, salable, and customized as compared to the permissionless blockchain. Likewise, in blockchain, the consensus algorithm is used to provide the data integrity and consistency across the nodes in the blockchain network. Over the last several years, many consensus algorithms have been developed, such as Proof of work (Pow), Proof of Stake (PoS), Delegated Proof of Stake (DPoS), Proof of Activity (PoA), Proof of Importance (PoI), Proof of Luck (PoL), Practical byzantine fault tolerance-based consensus (PBFT), and Raft. Table 1 summarizes the comparison of blockchain complexity based on the consensus algorithms.

In recent years, the evolution of the Internet of Things (IoT) [17,18,19] has revolutionized the way that people are living their lives. IoT is comprised of several thousand devices that monitor the environment, collect sensory information, and disseminate the information to some central location. Amongst the various applications, healthcare is one of the most emerging areas [20,21]. To maintain the health and fitness of individuals, doctors and physicians often recommend regular exercise. In this regard, individuals must spare their time for daily fitness activities and keep track of the appropriate diet plan to maintain better health. Like other applications of the IoT, some of the latest IoT devices [22,23,24] are available to track their fitness records. The Internet of Things (IoT) technology that is used to establish a connection among humans and peripherals is referred to as the Internet of Everything (IoE) [25]. The applications of the IoE include intelligent automotive, smart homes, smart fitness center, and smart vehicles [21,26,27,28], etc. In the case of a smart fitness center, several IoT sensor-based fitness wearables are planted in various parts of the trainee to acquire the fitness-related data to track individual fitness activities. The IoT plays a significant role in revolutionizing the fitness industry like the way trainers facilitate trainee training. Moreover, many critical issues are also addressed while using IoT fitness sensors, such as muscle imbalance, injury risks, and fitness time [29].

IoT plays an important role in the development of trainee by linking data from multiple fitness devices with enhanced analytics, which allow the trainer to process a massive amount of data in real-time. Furthermore, several IoT embedded fitness devices provide safety to the trainee, for instance, addressing muscle imbalance, managing workout plan, and notifying trainees when to take rest. These fitness embedded devices also keep track of each trainee activity while training. The IoT stipulates viable solutions for efficiently optimizing the productions in diversified domains. Despite having immense capabilities, the IoT suffers from various issues that are currently being addressed by the scientific community. The issues include big data analytics, security, connectivity, centralization, hardware capabilities, user data privacy, and GIS visualization, etc.

In recent years, the use of machine learning approaches has obtained wide acceptance in various applications [30]. These approaches are mainly used to apply data analytics to extract the hidden patterns and valuable information from the historical data to formulate and predict the future [31]. The data analytics allow users to consider latent patterns of the IoT data to provide Quality of Services. Because the IoT devices data are scattered over the servers in different formats and shapes, they are difficult to process using traditional approaches. Therefore, companies and users require a solution that can process data, and apply feature engineering on such a huge amount of IoT data [32]. According to Chung et al. [33], IoT applications are producing a considerable amount of data. The IoT applications exhibit poor performance when it comes to big data analysis. To date, the scientific community has presented different methods that rely on Artificial Intelligence (AI) based technologies, like Reinforcement Learning (RL) and Deep Learning (DL), to address the aforementioned issues [34]. The deep learning (DL) approaches have the potential to analyze massive amount of data for the tasks that require decision-making, as well as the prediction, classification, and detection of future demands in IoT. It provides the facility to extract features and scales the IoT big data gathered from multiple IoT devices. The combination of IoT and AI provides benefits in enhancing the performance of the IoT applications [35]. Lee et al. [36] presented an IoT model that is user-oriented and harnesses two types of approaches: (1) an uncertainty-driven arbitration and (2) bidirectional processing. The uncertainty-driven arbitration is utilized for big data analytic and the bidirectional processing is accountable for human knowledge and and communication networks.

The fitness data of the gymmers are of significant importance, and the integrity of this information is ultimately a big requirement during the the fitness training. In storing and processing of information on the Fog/edge, one question may arise whether the data has not been tampered, altered, or falsified due to any reasons. The information can be altered/modified according to the service providers interest, so this information may not be reliable. Therefore, there is a need to verify that information has not been altered/tampered. Recently, blockchain-based solutions have been introduced to provide various security services [18]. Like other applications, the Blockchain also offers a solution to verify the trustworthiness of information.

Nowadays, many researchers are using blockchain in big data analytics for data validation. At present, blockchain technology is a well-hyped innovation amongst researchers and, with time, it will become more popular and be widely adopted in several fields, from e-commerce to healthcare and image processing [37]. Since blockchain was developed in 2008, it has revolutionized the way that we automate transactions as well as deal, track, and trace payment logs [38]. Blockchain can perform effectively by eradicating the demand of central management to verify and govern the transactions and transfer control authority to every network participant to authenticate transactions. In the blockchain, every transaction is only signed and verified by the authorized and authentic participant of the network. Each transaction in the blockchain is secure using cryptographic hash algorithms, where mining nodes verified and signed the transaction and also maintained the entire ledger replica, which consists of transaction chained blocks [20,21,39,40,41,42]. This blockchain feature establishes a shared, synchronized, and secure record along with the timestamped value that is impossible to alter.

Currently, another eminent domain that is gaining attention is machine learning, which is used to learn, infer, and comply based on acquired IoT data. Similarly, blockchain technology also plays significantly in the field of computer science by controlling and wielding raw data. The incorporation of unclean IoT sensor data, for instance, missing data fields, redundancy, and incorrect data format, is considered to be the biggest challenge in the data science field. Using the help of blockchain-based smart contract, the data can be easily validated for data analytics.

The main contributions of this paper are contemplated below:

- The main aim of this research is to propose an enhanced smart contract based intelligent fitness service in blockchain networks.
- The proposed intelligent service model is based on an enhanced smart contract enabled relationship and a real-time inference engine that is used to infer new knowledge from the IoT environment and store the mined knowledge into the blockchain ledger.
- The proposed system is based on a permissioned blockchain model, where the IoT device information is secured and only authorized users can access the system logs and transaction history.
- The proposed blockchain model is a lightweight solution where the interactive client application uses the RESTful API to communicate between the IoT devices and blockchain network. The inclusion of RESTful API improves the system performance by providing the data offloading computation functionality.
- The proposed work also develops a prototype application for an intelligent fitness service, which demonstrates the strength of the proposed IoT blockchain architecture.
- The intelligent fitness service investigates the fitness data to recommend the diet and workout plan to the trainee.
- FInally, the robustness and effectiveness of the intelligent fitness service is evaluated using the Hyperledger Caliper in terms of latency, throughput, and resource utilization. The obtained results speak about the efficiency of the proposed system.

The rest of the paper is organized, as follows: a brief overview of the related work is presented in Section 2. In Section 3, we present the conceptual design of the proposed IoT blockchain architecture with a detailed description of the selected case study. A detailed discussion of the experimental setup and implementations is provided in Section 4. Section 5 contemplates the predictive analytics for secure fitness service and the discussion regarding the performance analysis is outlined in Section 6. Finally, we conclude this paper in Section 7 with an outlook toward our future work.

## 2. Literature Review

In this section, we discuss the existing work related to blockchain that is integrated with artificial intelligence and IoT. We also explain how modern technology, such as blockchain with artificial intelligence transform IoT. Blockchain technology provides a distributed and decentralized platform for IoT applications. In contrast, artificial intelligence is used for processing and analyzing the data in the IoT based applications, provides, decisions making functionalities and intelligence for the device to human. Moreover, we summarize the state-of-art existing studies in Table 2 and Table 3.

### 2.1. Blockchain and Artificial Intelligence in IoT

During the past few years, many researchers have published and addressed the research problem of artificial intelligence in blockchain, blockchain in artificial intelligence, and IoT and blockchain using artificial intelligence. Atlam et al. [35] presented an overview of the IoT and artificial intelligence along with opportunities and benefits in different artificial intelligence-based IoT application. Moreover, the author also described the blockchain technology, which is categorized into four sub-categories, i.e., blockchain characteristic and its taxonomy, blockchain application, consensus algorithm, and existing challenges. Wright et al. [43] presented a smart contract that is based on smart edge using the Ethereum platform. The defined platform enables offload calculation on nodes in a reliable way to verify edge devices in the trade of payment. Salah et al. [44] investigated the robustness and effectiveness of blockchain-enabled artificial intelligence along with open challenges that utilize artificial intelligence in the blockchain. The author also demonstrates the blockchain applications along with open issues while targeting artificial intelligence. Qian et al. [45] investigated the IoT based on three layers, such as the network layer, perception layer, and application layer, where blockchain technology provides the security for several open research and IoT devices problems. This system also addresses abnormal network traffic monitoring using identity verification and machine learning algorithms. Kshetri et al. [46] divide IoT platform based on four categories, i.e., capacity and costs constraints, server unavailability and cloud server down-time, deficient architecture, and susceptibility to manipulation. Nowadays, many IoT platform integrated with artificial intelligence and blockchain lacks several issues, like privacy, low latency, low accuracy, centralization, and a massive amount of data. Rathore et al. [47] demonstrate the IoT platform security architecture to provide scalable and secure IoT data to IoT platform in a decentralized manner. This system solves the issue of data centralization in IoT network. Rathore et al. [34] present the data security model using a deep neural network-integrated with blockchain in an IoT platform. The investigated platform enhances system performance in terms of accuracy and latency. Likewise, Atlam et al. [35] describe the effectiveness and robustness of IoT and artificial intelligence, which improves the system performance in terms of operating efficiency. The presented work is based on IoT application.

Most of the studies mentioned above are focused on blockchain that is integrated with artificial intelligence. However, these studies have many shortcomings in terms of latency, security, scalability, privacy, and throughput. Table 2 summarizes the state-of-arts comparison of existing studies with several technological aspects, such as efficiency, smart contract, access policy, functionality, crypto-currency, and consensus algorithms.

**Table 2:** Critical analysis of existing studies related to Internet of Things (IoT) blockchain and artificial intelligence.

Name	Year	Smart Contract	Technological Aspects	Consensus	Access Policy	Crypto-Currency	Functionality
Rathore et al. [34]	2019	Yes	Blockchain+AI	Complete Nodes	Not Defined	Yes	BlockDeepNet
Rathore et al. [47]	2019	Yes	Blockchain+AI	Complete Nodes	Premissionless	No	Security Architecture for IoT Network in Smart City
Salah et al. [44]	2019	Not Defined	Blockchain+AI	Complete Nodes	Premissioned/ Permissionless	Not Defined	Healthcare, Microgrid, Farming, Ocean exploration, Banking
Atlam et al. [35]	2018	Not Defined	IoT+AI	Complete Nodes	Not Defined	Not Defined	IoT Platform
Wright et al. [43]	2018	Yes	Blockchain+IoT +Edge Computing	Complete Nodes	Premissionless	No	IoT Platform
Qian et al. [45]	2018	No	Blockchain+IoT	Complete Nodes	Not Defined	No	Security Architecture for IoT Network
Kshetri et al. [46]	2017	No	Blockchain+IoT	Complete Nodes	Not Defined	No	IoT Platform
**Proposed** **Solution**	**2020**	**Yes**	**Blockchain+AI** **+IoT+** **Inference** **Engine**	**Arbitrary** **Nodes**	**Permissioned**	**No**	**Intelligent Fitness** **Service Based on** **IoT Blockchain** **Platform**

### 2.2. Blockchain in Fitness

Over the past several years, many electronic devices have assisted humanity in sharing, digitizing, and presenting fitness-related data to both the trainer and trainee. In this section, we will investigate a few fitness applications based on blockchain.

Elliott et al. [48] presented an incentive-based application that encourages users to perform physical activities. This work aims to demonstrate and measure the change in the user physical activity, such as daily step count. Derlyatka et al. [49] proposed a sweatcoin that acts as a digital currency that is used as an incentive provided by the user in exchange for the physical activity. The goal of this is a startup is to use user personal physical data in the form of step-count and return, provide an incentive in the form of sweatcoin. In 2018, a Switzerland based startup head by Joseph Anthony developed an application that monitors user physical activity. The startup is named as Run2Play, which aims to incentivize users for physical activity [50]. In 2017, Martin presented an Ethereum startup, named Movement app, which aims to incentivize users for physical activities, such as jogging and running, while using a treadmill and any outside physical activity. The startup also built a market place where the user of the movement app can use their token to purchase fitness gears and nutrition, as well as register for online fitness and yoga courses. Moreover, the user of movement app can also exchange the token into other cryptocurrency [51]. In 2016, Jaroslav developed a fitness application that aims to educate people on how to exercise properly. The startup is named as Truegym, which is Ethereum based uses machine learning approaches that analyze fitness data acquired from trainer and devices to recommend training plan for every user [52]. In 2018, Drake designed a fitness application named as The Hustle. The Hustle is an Ethereum based blockchain application aims to promote wellness, health and fitness and also incentivize for staying healthy. The startup develops an Ethereum token that is rewarded to each user who participates in fitness-related activities. The HUSL token is transferred to each users application builtin wallet, which can be easily exchanged into other currencies, such as, ETH, BTC, or USD [53]. Table 3 presents the contemporary fitness application based on blockchain.

**Table 3:** Critical analysis of existing studies for fitness service.

Authors	Year	Approach	Platform	Objective	Limitations
Joseph Fargnoli and Chelsey Clime	2018	Run2Play [50]	Ethereum	Store health fitness data and incentivize user with RUNtoken. Gaming application based on augmented reality utilizing RUNCoin proof-of-fitness, and proof-of-stake in order to reward users for fitness activity.	Low Scalability. Hosted on public server. Required high computation.
Martin Holt	2017	Movement [51]	Ethereum	Incentivize users for physical activities, such as jogging, running, using a treadmill and any outside physical activity	Low Efficiency. Less secure. Low scalability. Required mining
Jaroslav Štreit	2016	Truegym [52]	Ethereum	Truegym is Ethereum based uses machine-learning approach that analyze fitness data acquired from trainer and devices to recommend training plan for every user. Incentivize users with TGC Token as a reward in exchange for physical activity.	Low Efficiency. Less Secure. Low scalability. Required mining
Kristopher Floyd	2018	TeamMate [54]	Ethereum	Store healthcare data such as vital sign and fitness data such as users physical activities. Incentivize user with TMT token through consensus algorithm via smart contract.	High energy consumption. Less secure. Low Scalability. Required mining
Bryan Seiler	2018	Fitrova [55]	Ethereum	Fitness application aims to store fitness user data. Develop FRV a token which provides concise and clear payment, secure and lightweight payment transferring platform.	Low throughput. High energy consumption. Less secure. low scalability
Drake Blankenship	2018	The Hustle [53]	Ethereum	Promoting wellness, health and fitness. Incentivize user for staying healthy.	High latency. Low efficiency. High power consumption. low scalability.
Daniel Sanchez	2019	180NF [56]	Stellar	Recommend wellness, workout, diet and nutrition. Scheduling personalized exercise and training. Incentivize user with a token in exchange of data.	Less Scalability. low throughput. High power consumption.
Robert Maxwell	2017	FIT Token [57]	Ethereum	Blockchain-based sport and fitness an application that allows the user to use FIT token to buy memberships and make a booking at sport and fitness entertainment.	Required high energy consumption for token mining. less scalable. low performance efficiency.
Jean-Michel Alfieri	2019	StepChain	Ethereum	A responsive fitness application reward users in exchange for physical activities and calories burned. Acquired data from built-in smartphone sensors. Introduced StepCoins.Track fitness progress.	Required high mining cost. less scalable. Less secure. High latency.
**Proposed System**	**2020**	**Intelligent** **Fitness Service**	**Hyperledger** **Fabric**	Intelligent fitness service based on smart contract enabled inference engine. The real-time inference engine derived new body composition function from the user and device network. The blockchain platform also recommend diet plan, and fitness plan for trainee. Moreover, the system also predict the future diet plan and workout plan.	**Limited network size.**

As aforementioned, these blockchain-based platforms are either not permissionless or open-source; hence, the general user is unable to upgrade or modify the existing system for their purpose. Moreover, the majority of the methods presented in the literature review are related to IoT and blockchain with theoretical knowledge of artificial intelligence, or they incentivize user with a token for providing fitness data. Nonetheless, none of any previously presented systems use real-time inference engine to drive body composition function using a permissioned blockchain platform that is known as Hyperledger fabric. Furthermore, most of the existing fitness applications discussed above use the inherent crypto-currency, which decreased the performance of the system in terms of computational power during the transaction. To the best of the authors’ knowledge, there has been no functional smart contract centric relationship and inference engine model developed for deriving body composition from IoT fitness environment.

## 3. Proposed Relationship and Inference Mechanism of Smart Contract Based on User and IoT Device Profile

### 3.1. Intelligent Service Model Based on Enhanced Smart Contract

**Figure 1:**
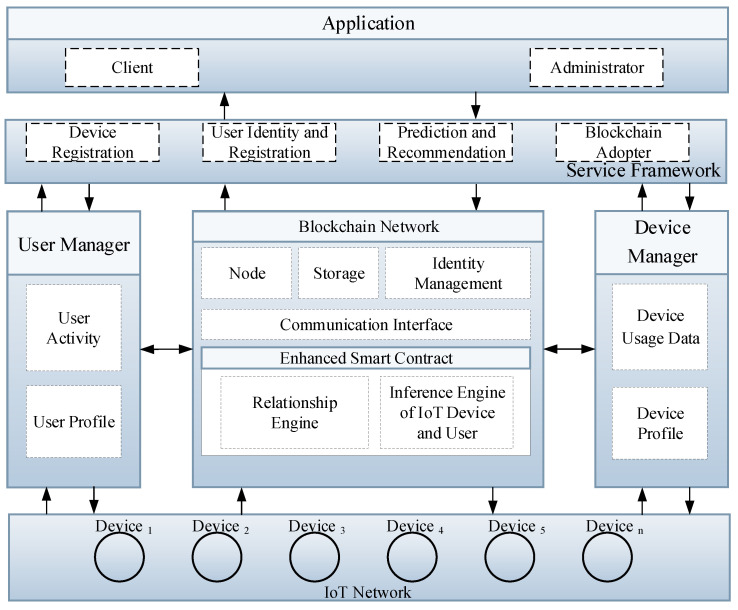
Proposed intelligent service model based on enhanced smart contract with relationship and inference engine in IoT network.

The Internet of Things (IoT) comprises of devices that generate, process, and exchange huge amount of critical data as well as privacy-sensitive information. To assure user privacy, a light-weight, transparent, high-throughput, and scalable blockchain IoT platform has been introduced to safeguard users’ privacy. Figure 1 presents the intelligent service model that is based on enhanced smart contract with a relationship and inference engine in an IoT network that consists of four modules, such as application, service framework, blockchain network, and IoT network. The application module comprises of a client and administrator whose responsibility is to interact with the front-end using the Representational State Transfer Application Programming Interface (RESTful API). Similarly, the service framework is responsible for services that are related to device registration, user identity, user registration, recommendation, and blockchain adopter, etc. These services are offered by intelligent IoT blockchain architecture, which performs user and device management. The user and device manager are used to acquire data from the IoT and user network. The data that are gathered from the user manager include user activity and user profile, whereas the device manager manages data related to the device profile and device usage data. The intelligent blockchain smart contract consists of relationship and inference engine, which is used to calculate and infer new knowledge from the user and device manager. The data from the user and IoT network are stored into the blockchain and visualized to the client application using the blockchain adopter. Finally, the blockchain network is a distributed ledger technology consisting of several peers that are used to track, authenticate, and execute the set of transactions in a Peer-to-Peer network. The distributed ledger is a shared replica of data that are available across the blockchain network where all of the participants of the network can have the same copy of the ledger.

Any modifications to the ledger are reflected in all copies across the entire blockchain network. The smart contract in the blockchain is a chaincode that is triggered by the clients through client application to access and modify the ledger. The smart contract is installed and initiated on each peer in the blockchain network. The identity manager provides the authentication and authorization, where only valid participants can join the blockchain network. The application layer provides various services and visualizes meaningful data from physical devices.

### 3.2. Intelligent Service Architecture Based on Enhance Smart Contract

**Figure 2:**
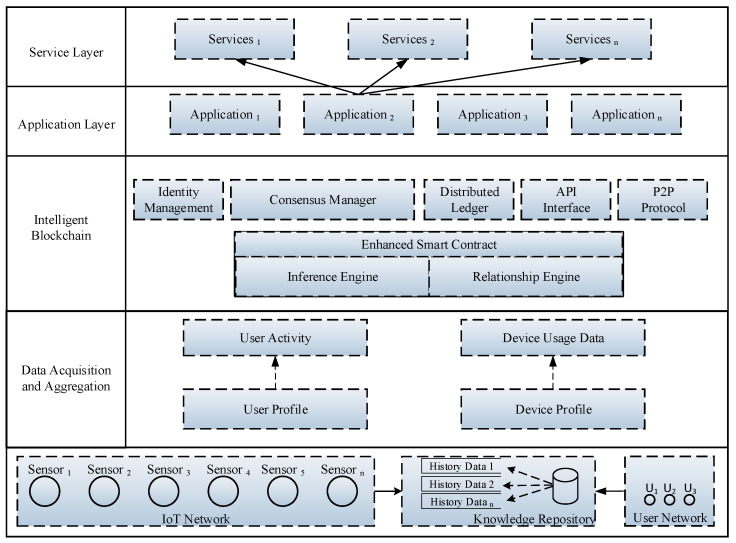
Proposed intelligent service architecture based on enhanced smart contract with relationship and inference engine in IoT network.

Figure 2 presents the proposed intelligent service architecture that is based on enhanced smart contract, which is comprised of five layers, such as service layer, application layer, intelligent blockchain layer, data acquisition and aggregation layer, and physical layer. The proposed intelligent service architecture is based on modular design where each layer is decoupled from other layers that provide ease to the developer to modify or replace the existing modules without a fallout of the entire system. The physical layer consists of IoT and user network where historical data are stored in the knowledge base. The data acquisition and aggregation layer acquires the data from the IoT and user network. Moreover, this layer also performs some additional data functionalities, such as statistical analysis and elimination of redundant data.

The intelligent blockchain layer consists of various services, such as identity management, consensus manager, distributed ledger, API interface, P2P protocol, and smart contract functionality. The identity management is used for identifying, authorizing, and authenticating the users to have access to the proposed IoT blockchain application by associating user rights along with established identities. The blockchain is distributed database technology where each block contains a set of transactions which are cryptographically secure with different kinds of encryption algorithms. Each node in the blockchain contains a replica of the ledger, where any change in one copy will update other copies of ledger across entire blockchain within the second to minutes. The events in the blockchain are triggered based on the successful execution of the smart contract, which results in the creation of a new block in the chain of network. The member service provider allow the users to participate in the blockchain network by issuing the identity certificate approved by the network members. The enhanced smart contract comprises of inference and a relationship engine, which are used to calculate and infer new knowledge, useful patterns, and obtain hidden insights from IoT devices and user network data. Lastly, the application layer offers numerous services and interfaces that are provided by the service layer in order to expose the functionality to the client applications.

### 3.3. Interaction Model of Proposed Intelligent IoT Blockchain Platform

**Figure 3:**
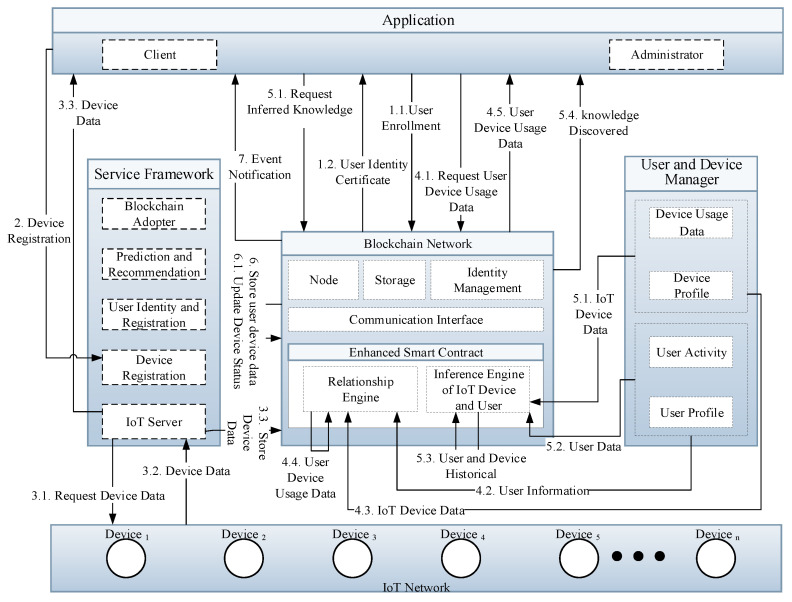
Detailed intelligent service configuration based on enhanced smart contract enabled relationship and inference engine in IoT network.

Figure 3 presents the proposed intelligent service configuration based on an enhanced smart contract in the IoT network. The designed intelligent service model is not limited to technical infrastructure, but also a user service framework that exposes a smart contract and distributed ledger as a service to the client application. The client application provides a perceptive interface that supports intuitive services, like user enrollment, IoT device registration, and user-device data calculation, which is used to submit transaction proposals to the blockchain network. The user enrollment is mandatory before submitting the transaction proposal, which generates a private secret key that is used to sign the transaction. The transaction is the process of reading and writing IoT device data from the ledger that execute across the entire blockchain network. The system users, like admin, can submit the transaction like register a new IoT device, user profile management, relationship engine, or generate a new task via IoT server. Afterwards, the IoT server transfers the request to the blockchain to perform specific tasks. The IoT server also transfers the task request from client application to the IoT devices and sends back the response, like personal user data, to the client application in real-time. Because the blockchain platform provides authentication and authorization to the participants, the related transactions can be directly executed by the specific participants. The IoT data status, for instance, in the case of smartwatch, the heart rate data are stored into the ledger and notify to clients based on the threshold defined in the smart contract. The notification is sent to the client application if the value exceeds the verge level. The enhanced smart contract consists of a relationship and inference engine that is used to calculate and derive additional knowledge and features from the IoT device and user network data.

### 3.4. Execution Flow of Proposed Intelligent IoT Blockchain Platform

**Figure 4:**
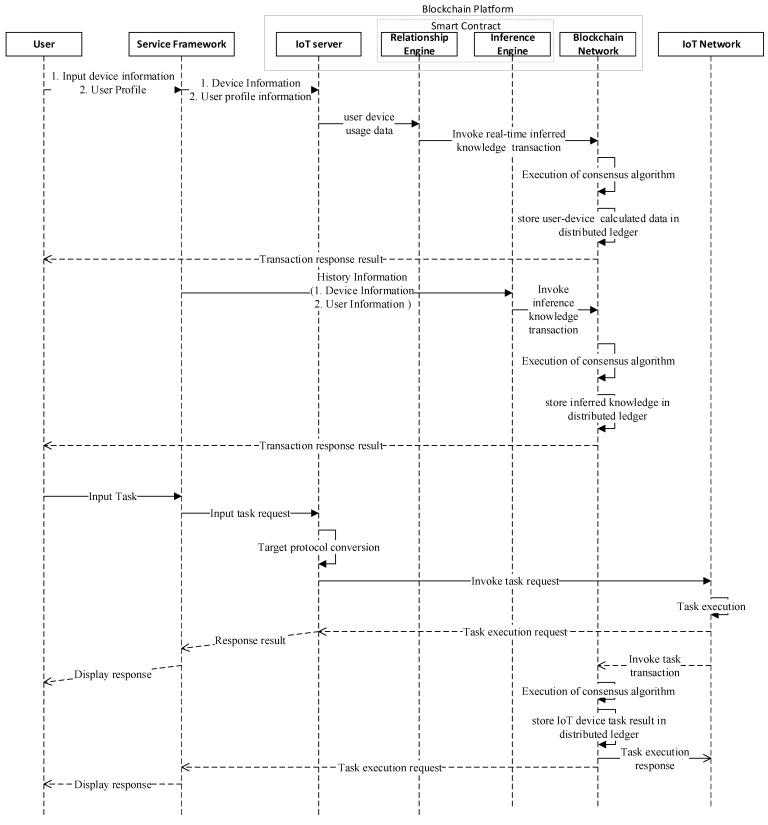
Sequence diagram of intelligent service based on enhanced smart contract enabled relationship and inference engine in IoT network.

Figure 4 shows the workflow of intelligent service based on an enhanced smart contract enabled relationship and inference engine in IoT network. The system user is allowed to connect and employ network provided services. The system user inputs the information that is related to IoT devices and users, like device profile, device usage data, user profile, and user activity from the client application. The information is sent within the request header where the request is forward to IoT server, which, in return, trigger different services, like device registration, user-device calculation, and inference engine defined in the enhanced smart contract. The relationship engine is a chain code that is defined in the smart contract to deduce real-time inferred knowledge that is based on the currently entered data. Afterwards, the execution of the consensus algorithm starts in the blockchain network, where the real-time inferred knowledge is stored in the state database and every network peer affixes the transaction into the blockchain network. The response notification of successful transaction which updates the ledger state is sent the client. Furthermore, the enhanced smart contract also supports inference engine functionality where the new knowledge is inferred from the historical data of the user and IoT devices, as defined in the smart contract. Likewise, the system user can also invoke some task-related services, like acquired data, from the IoT devices and send back the response transaction result to the IoT server, from where it was visualized to the client application through Http protocol. The successful transaction is stored in the blockchain network and updated into the ledger state by every peer.

## 4. Intelligent Fitness Service Model Based on Relationship and Inference Engine

**Figure 5:**
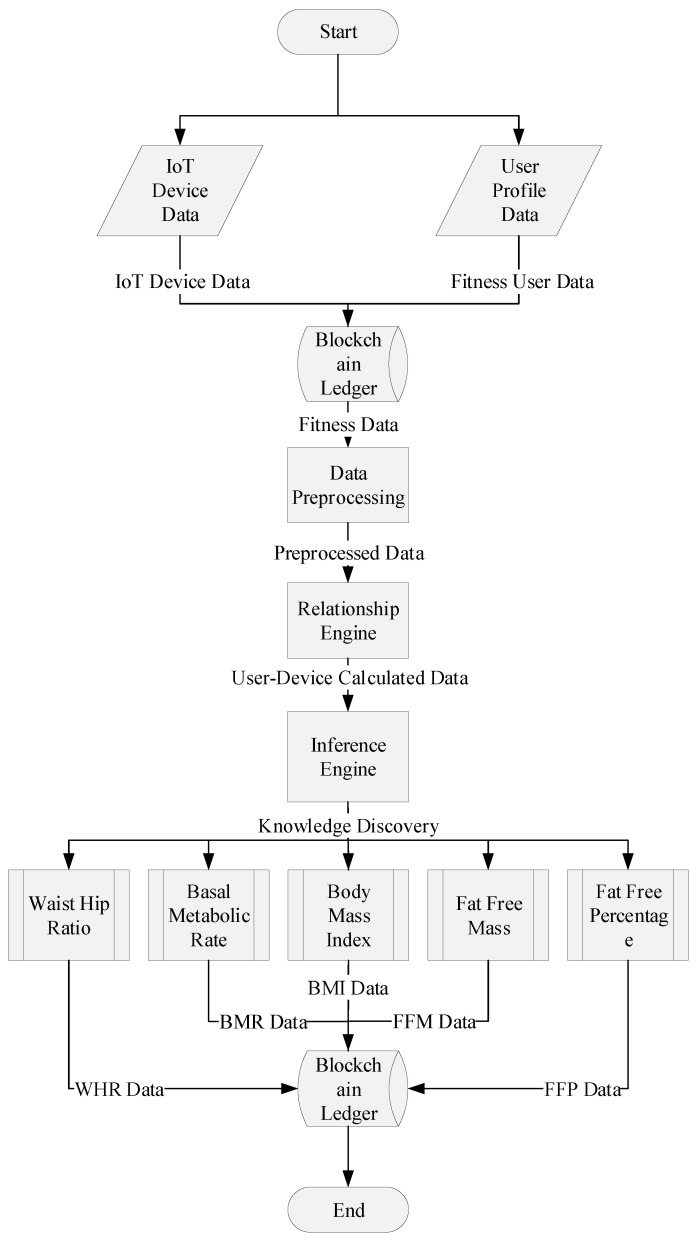
Flow chart of intelligent fitness service model based on enhanced smart contract enabled relationship and inference engine in IoT network.

**Figure 6:**
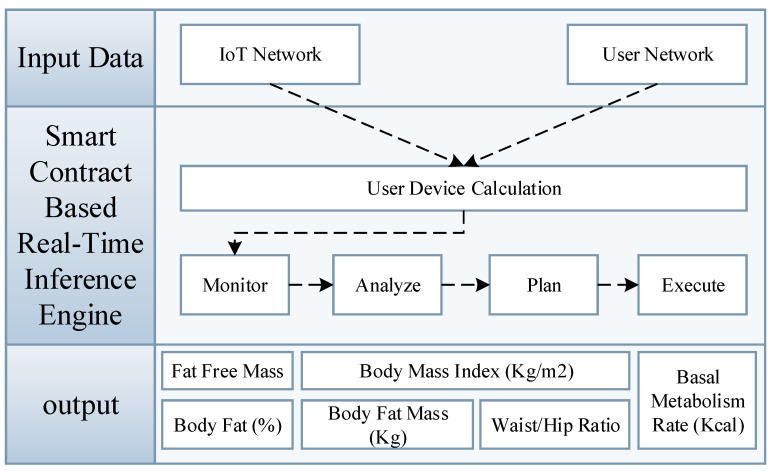
Enhanced smart contract for intelligent fitness service based on real-time inference engine.s

Inference refers to deriving additional knowledge from already known facts. In machine learning, the dataset provides base knowledge, whereas the machine learning algorithms traverse the data for different patterns to form some general rules that are inherent in the data. The rules are then applied to the new data to infer new knowledge that is not part of the data, but satisfies the rules. For this, different methodologies are presented to code rules and decode them for new data. Fuzzy logic is one unique technique being widely adopted to infer knowledge from the data using fuzzy set techniques. Different rule engines, such as Drools and alike, are also introduced whose job is to form optimal rules based on the patterns within the data and applies the rules to infer new knowledge from the data. The flow of intelligent fitness service model based on relationship and inference engine is also defined in flow chart as shown in Figure 5. In the proposed intelligent fitness blockchain platform, we introduced an enhanced smart contract-based inference engine which uses real-time IoT fitness device and user profile data to infer new knowledge, as presented in Figure 6.

**Figure 7:**
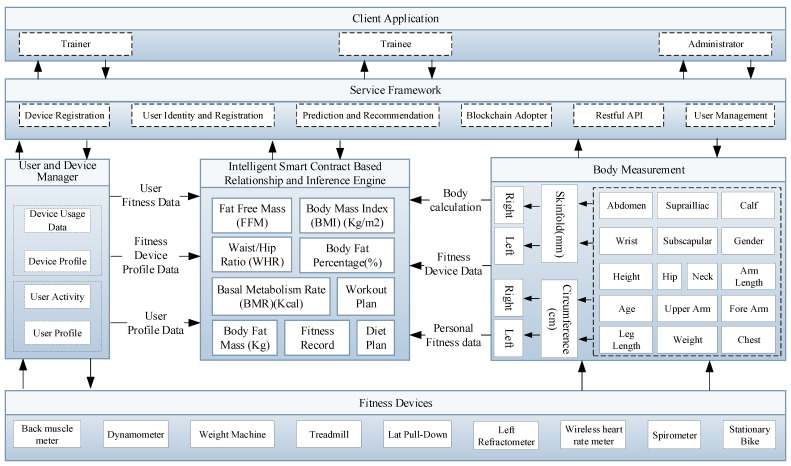
Proposed intelligent fitness service based on enhanced smart contract enabled relationship and inference engine in IoT network.

The body measurements data thata re based on the user profile and IoT fitness device data are used to derive new knowledge, such as *Fat-Free Mass* (*FFM*), *Body Mass Index* (*BMI*), *Body Fat Percentage* (*BFP*), *Body Fat Mass* (*BFM*), *Waist/Hip Ratio* (*WHR*), and *Basal Metabolism Rate* (*BMR*), as defined in the smart contract, as shown in Figure 7. The *BMI* calculation is used to determine body weight status, i.e., overweight, normal, or underweight, as presented in Table 4. The *BMI* is computed using the following Equation (1).

(1) $$Body\;Mass\;Index=\frac{weight\;(Kg)}{height^{2}\;\left(m\right)}$$

where *weight* and *height* is acquired from IoT fitness device and user profile.

Similarly, *BFP* is also called as adipose tissue in the form of lipids. There are many ways to calculate the *BFP*; however, in the proposed intelligent smart contract-based inference engine, we compute the *BFP* using Equation (2).

(2) BodyFatPercentage=α×BMI+β×Age−γ

where α, β, and γ are constant, where the values depend on the input data, i.e., user and fitness device data. According to the American Council on Exercise, the value of α and β is 1.20 and 0.23, respectively, for both male and female. However, the value of γ varies in case of male and female, e.g., (Male = 16.2, Female = 5.4). Table 5 summarized the *BFP* range for both male and female.

Likewise, the *FFM* is one of two human body constituents, i.e., fat and rest body. The healthy *FFM* is similar to *BFP*, which is presented in Table 5. The *FFM* of the body is measured using the following Equation (3):

(3) $$Fat\;Free\;Mass=weight\;\left(Kg\right)\times \left(1-\frac{BFP}{100}\right)$$

where *BFP* is taken from the Equation (2), and weight data are acquired from the user profile.

*WHR* is used to calculate the dimension ratio of the circumference of the weight over the hip. *WHR* is used to identify several factors that are related to the health, such as asthma, heart disease, and other health issues. The usual range of *WHR* for the male is less than 0.90, whereas, for female, the normal range is less than 0.85. The *WHR* is simply defined as the ratio of waist over the hip.

Finally, *BMR* is used to calculate the amount of calories per day required by the human body while at rest. The *BMR* is computed using the following Equation:

(4) $$Basal\;Metabolism\;Rate\;\left(Kcal/day\right)=K\times weight\;\left(kg\right)+\omega \times height\;\left(cm\right)-K_{1}\times age\;\left(year\right)+\epsilon \;\left(Kcal/day\right)$$

where ϵ is set based on gender data, e.g., (Male: +5, Female: −161). Similarly, *K*, ω, and $K_{1}$ are the constants with the values 10, 6.25, and −5, respectively.

The output of the proposed fitness blockchain platform is derived from the fitness devices, user profile, and user-device usage data using smart contract-based real-time inference engine. The user device data calculation is monitored, analyzed, planed, and then executed while using the mathematical model that is defined in the smart contract.

## 5. Development of Intelligent Fitness Services Based on Enhanced Smart Contract Enabled Relationship and Inference Engine in IoT Network

The implementation of the proposed system is divided into five sub-components, i.e., intelligent fitness blockchain network, fitness IoT server, IoT gateway implementation, fitness blockchain front-end development, and predictive analytics model. The implementation and experiments of the proposed platform are conducted on eighth-generation machine equipped with Intel core i-5 processor along with 8 GB memory and Ubuntu Linux 18.04 LTS. For blockchain development, the docker engine 18.06.1-ce and docker composer 1.13.0 versions are used, which provide the development environment to set up the container and docker image on the virtual machine. The docker composer provides the run-time environment for the docker engine. Furthermore, we have used Hyperledger Fabric, an open-source framework that is hosted by Linux Foundation, which is used for client software toolkit (SDK) using Node 8.11.4 version. The composer web-playground is a web interface used to develop smart contract using a component of the business network archive (BNA). The database that is used for the back-end of the blockchain network is DBcouch that determines the current state of the ledger. The composer command-line interface (CLI) is used to perform smart contract management. Finally, the business logic of the proposed system is exposed to front-end GUI using the REST API, which is generated through the composer REST server. Similarly, the technologies and development environment for the implementation requires IoT fitness device server that resides inside Raspberry Pi, which acts as an IoT gateway. Moreover, we have installed Android Things on the Raspberry Pi to provide Java language programming support. The MQTT communication protocol is used to communicate between the IoT server and the device server, whereas HTTP is used for communication between blockchain and device server. Physical devices (such as Spirometer, Stationary Bike, Wireless heart rate meter, Treadmill, Dynamometer, and fitness watch, etc.) are abstracted into MQTT resources as components of the server. The server identifies each resource with the unique URI. For the front-end of the blockchain-based intelligent, personalized fitness data safety platform, we used multiple programming languages, such as JavaScript, Cascading Style Sheet (CSS), and HTML. Moreover, we also used open-source web development toolkits, such as jQuery and Bootstrap. Lastly, in order to implement the predictive analytic model, we have used PyCharm Professional 2020 as an IDE with Python programming language. Deep neural network and support vector regressor are utilized to implement the prediction of the workout and diet plan. We have used jQuery plug-in Notify.js for generating a customizable notification to the end-user. The end-users can subscribe the blockchain services by submitting a transaction through REST API using the HTTP communication protocol. Table 6 summarizes the details regarding the complete development environment for the intelligent fitness service (based on enhanced smart contract enabled relationship and inference engine in IoT network).

### 5.1. Use-Case Implementation and Deployment

The implementation of the proposed intelligent fitness service is visualized in Figure 7. The implemented case study is based on blockchain-based intelligent fitness service, where the trainee is equipped with multiple fitness devices, such as back-muscle meter, dynamometer, weight machine, lat pull-down, treadmill, spirometer, stationary bike, wireless heart-rate meter, and left refractometer, etc. The proposed platform is capable of establishing a connection between the IoT fitness devices, IoT server, blockchain network, and the predictive analytic model. The Raspberry-pi behave as an IoT gateway, which is used to route fitness data to fitness IoT server. Similarly, the fitness IoT server is used to process fitness data request and yield the user fitness data to the users through the blockchain network. The IoT server provides the services, but, not being limited to the data penetration, effectively furnishes the data in order to visualize to client end, and checking the data threshold value. The proposed system uses Hyperledger fabric with four peers and one orderer node in order to implement the blockchain network. The peers and orderer resides in the docker container as an image. Every peer in the network contains a smart contract and storage for data to write a transaction block to ledger. CouchDB is the database used in the proposed blockchain platform, which acts as a state database providing rich queries, where the JavaScript Object Notation (JSON) is used to model the smart contract data. The data stored in the state database are in the form of key-value pair as well as also in multiple key-value pair. Likewise, the REST server supports several RESTful APIs that expose the back-end functionalities of blockchain to the front-end, as defined in the smart contract. These RESTful API services can be accessed by either the IoT fitness devices or front-end client application. Moreover, the RESTful API also host Fabric client, which uses the Google remote procedure call (gRPC) system in order to communicate with the Fabric network. The real-time inferred knowledge is computed using the smart contract, which includes, but is not limited to, *FFM*, *BMI*, *BFM*, *WHR*, *BFP*, and *BMR*. Moreover, the smart contract also supports the inference engine based on historical data of IoT fitness devices and user information. Finally, the predictive analytic model predicts the workout plan and diet plan for the trainee fitness management, which helps the fitness centre to recommend healthy diets for the trainee to enhance fitness habits, nutrition, lifestyle, and health. Furthermore, it will also help fitness owners to learn more about their members to make better future decisions, such as creating different advertising campaigns that are more effective.

Finally, all data are stored according to the timestamp, where any changes in the state database are reflected across the whole blockchain network. The consistency of every ledger copy is maintained and ensured by the consensus algorithm (i.e., PBFT considered in this research), which is implemented in the orderer node. The main purpose of the orderer node is to order the transaction based on first come first serve basis in the entire blockchain network. Finally, the client-end is notified by emitting a notification from the blockchain network while using Web-Sockets.

### 5.2. Smart Contract Modeling of the Case Study

In the proposed fitness blockchain platform, we have designed an intelligent smart contract system that is based on the inference engine using the Hyperledger Composer. The Hyperledger Composer is an open-source toolkit and framework used to develop blockchain-based applications. The key concept of thge Hyperledger composer is Business Network Definition, which is used to create a Business Network Archive (.bna) file. The .bna is composed of model file (.cto), script file (.js), access control (.acl), and query file (.qry). Furthermore, the .cto file is divided into transaction, assets, and participant. Table 7 summarized the details of the transactions, assets and participants along with the components and description. In the designed system, the participants are the users of the business network, such as admin, trainee, and trainer, whose responsibility is to modify the assets and submit transactions.

Similarly, the assets are the goods, properties, and services that are stored in registries. In the proposed system, the assets are body measurements, features, work-out plan, fitness goals, diet-plans, and fitness device records. Finally, the transactions are also part of the smart contract which are used to interact with the assets. Through client application, the transactions are invoked by the participants of the business network to modify assets across multiple blockchain networks. Several transactions are defined, which are based on inference engines, such as *FFM*, *BFP*, DMI, *WHR*, and *BMR* as defined in Figure 6. Similarly, the .js file contains the transaction processor function, which is a combination of logical operations using the inference engine that is defined in the smart contract. *FFM* is a transaction function, which is used to calculate the body fats, based on the data that are acquired from the IoT fitness devices and user profile information, also defined using Equation (3). In the smart contract, the transaction processor functions are used to update the value of the assets in the registry. Moreover, we also defined queries as a part of the smart contract, being mainly written in the bespoke query language. Queries can be used to provide ease in extracting data from the blockchain network. The query structure is divided into several components, such as description and statement. The descriptions are the string that describes the function of the query, whereas the statement is the logical operator that controls the behavior of query.

The proposed fitness blockchain platform also supports RESTful application programming interface (API), which was developed and generated using a composer-rest-server. The main aim of RESTful API is to connect IoT device, web interface, and the blockchain network. Moreover, the RESTful API is based on HTTP request, which comprises of media-type and base URI that represents a data element state transition, such as Application/Json and verbs, e.g., POST, GET, DELETE, and PUT. The verb indicates the action that is performed on the request, whereas the URI implies the data entry path. In the designed fitness blockchain platform, a Get request to the resource URI (e.g., /api/FitnessDevice) would return the fitness device information. In contrast, the POST request to the similar resource URI will store the data that are enclosed in the request header packet. Table 8 shows the list of RESTFul API generated to expose the servers of a business network to the client application.

### 5.3. Execution Procedure of the Case Study

**Figure 8:**
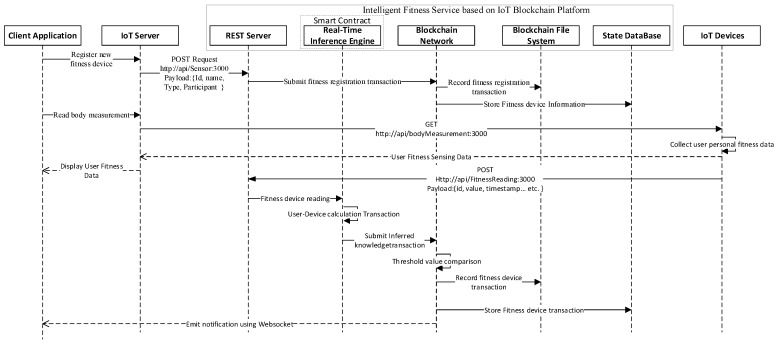
Intelligent smart contract based real-time inference engine.

Figure 8 presents the execution of the proposed intelligent fitness service based on IoT and blockchain. In the start, the participants of the system, such as the admin of the fitness centre, input the fitness device information through client application to register a new fitness device. The fitness device information is sent through an IoT server that processes and requests the REST server using the POST method. The payload request contains device information that is submitted to the blockchain network through fitness device registration transaction. The transaction is recorded in the blockchain file system and the device information is stored in the state database. Likewise, the system users, like Trainer, trainee, can request ReadBodyMeasurement transaction to IoT server, the request is sent to the specific IoT fitness devices that collects the user personal fitness data and visualizes the collected data to the client application through IoT server. Similarly, the IoT fitness data POST request is sent to the REST Server, where data are processed and passed to the inference engine.

The inference engine is used to derive additional knowledge, such as *FFM*, *BMI*, *BFM*, *WHR*, *BFP*, and *BMR* from the UserDeviceCalculation transaction along with the threshold detection. The inferred knowledge is recorded in the state DB using the blockchain network. All of the transactions triggered through client application are stored in the blockchain file system, and the response to the transactions is emitted through WebSocket in the client application.

## 6. Predictive Analytics Model for Secure Fitness Service

In this section, we use the IoT fitness data, apply pre-processing, and select the strongly correlated features and perform prediction while considering several renowned prediction algorithms. In this research, we acquired IoT fitness data from the fitness centre of Jeju national university, South Korea. The dataset is comprised of the following attributes: trainee name, along with other personal information, monthly Diet plan, monthly workout plan, and IoT fitness device information, such as device name, device type, model, etc. The acquired IoT fitness dataset consists of 100,000 personalized fitness record over the last eight years (2013–2020). The diet and workout plan is prepared based on the following fitness types, i.e., normal, obesity, and athlete, to maintain the health profile of fitness trainee.

The predictive analytic model in the proposed intelligent IoT fitness service consists of the following phases, such as data collection of IoT fitness data, data pre-processing, data normalization, training and testing machine learning-based prediction models, and performance evaluation. In the pre-processing data layer, the raw data are transformed into reliable data to discover hidden patterns and knowledge using deep learning approaches. The process of data pre-processing starts with data cleaning, which is used to remove duplication, blank space, change the text to upper/lower case, and spell-check. After cleaning, the next step is data integration, which is used to integrate the data from the various sources to provide the user with the unified view of data and format data for mining the patterns. Afterwards, data transformation is applied to the resultant dataset. Data transformation can be performed while using different approaches, e.g., feature construction, generalization, normalization, and smoothing, etc. In the proposed system, we performed normalization to scale the data of an attribute in a range between 0–1. In general, normalization is required when we have attributes with different scale, so normalization brings all of the attributes on the same scale. Missing values in data is due to a faulty sampling and acquisition process. The missing values can produce biased estimation, inaccurate statistical estimations, and sometimes invalid conclusions. In the proposed system, we fill the missing values using the probabilistic model that is based on maximum likelihood. Finally, the proposed system is assessed using various renowned machine learning-based classifiers considered in this study, which are decision tree (DT), logistic regression (LR), support vector machine (SVM), and K-nearest neighbours (K-NN).

The scalability and robustness of the proposed intelligent fitness service are assessed using trainee body composition functions. In the proposed system, we have computed body composition functions, which consist of the following features, such as *FFM*, *BFP*, *BMI*, *BMR*, and *WHR*. These features are used as inputs to the machine learning algorithms for recommending diet and workout plan. This system utilizes the IoT fitness dataset, which consists of labels that include diet plan 1, diet plan 2, diet plan 3, workout plan 1, workout plan 2, and workout plan 3. The (Diet plan 1, workout plan 1) is for regular and average trainees, (Diet plan 2, workout plan 2) is for obesity trainees, and (Diet plan 3, workout plan 3) is for athletes, as shown in Table 9.

**Figure 9:**
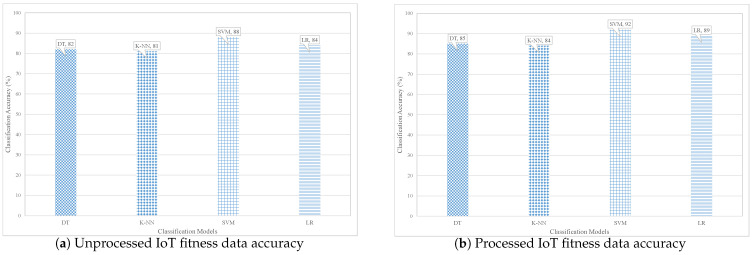
Performance results of different learning methods for IoT fitness dataset.

Furthermore, the fitness data are used to test different machine learning models, such as DT, LR, SVM, and K-NN. These learning models are trained and tested on the fitness dataset, where the data distribution ratio for the training and testing is 80:20. Ten-fold cross-validation is used to evaluate the learning models used in the proposed system. The accuracy of different machine model is evaluated using two scenarios, i.e., the accuracy with unprocessed data, and the accuracy with the processed data, as shown in Figure 9.

Several experiments were performed to investigate the performance of the contemporary ML approaches. The obtained results show that the SVM model outperformed K-NN, LR, and DT approaches by attaining 92% accuracy. The classifiers are trained and tested on the IoT fitness dataset and they are evaluated using 10-fold cross-validation.

Table 10, summarized the results of performance evaluation of the implemented model based on classification. It is estimated that the SVM classifier model outperformed DT, LR, and K-NN in terms of accuracy, precision, recall, and f-measure.

**Figure 10:**
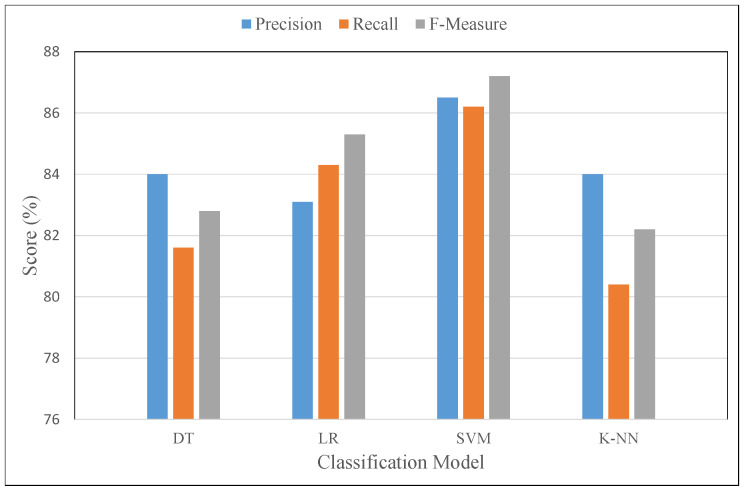
Performance evaluation using different classification models in terms of precision, recall, and f-measure.

Figure 10 visualizes the performance analysis based on different classification model, i.e., DT, LR, and SVM. Several performance metrics are used to evaluate the proposed fitness framework. It is evident from the graph that the SVM model performed well in terms of precision, recall, and f-measure, with scores of 86.5%, 86.2%, and 87.2%, respectively.

## 7. Performance Analysis

This section presents the performance evaluation and experimental results of the proposed blockchain-based Intelligent fitness service.

**Figure 11:**
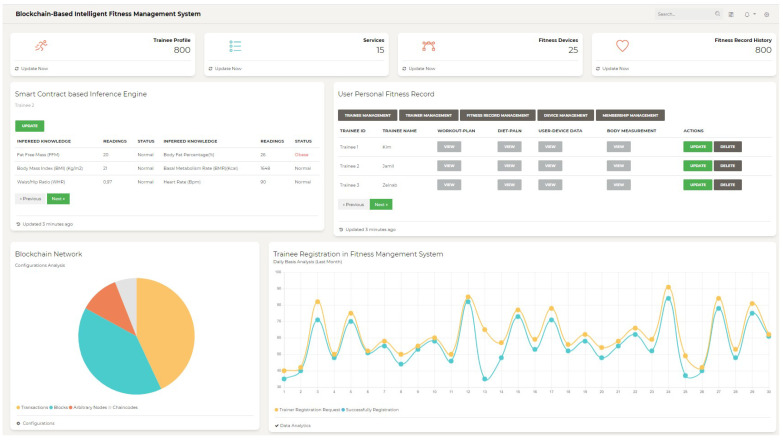
Intelligent fitness service front-end user interface.

The obtained results of the proposed Intelligent fitness service based on IoT blockchain platform are presented, as follows. Figure 11 shows the main dashboard of the proposed system, which shows the core functionality of the designed intelligent fitness service. The core functionalities include trainee profile, trainer profile, fitness services, fitness device profile, and fitness record history. Moreover, the dashboard also shows the smart contract enabled inference engine based body measurement calculator that includes, e.g., *FFM*, *BFP* (%), *BMI* (Kg/m^2^), *BMR* (Kcal), *WHR*, and Bpm. Furthermore, the dashboard also includes information that is related to user personal fitness record, along with trainee management, trainer management, fitness record management, device management, and membership management. The user personal fitness record for every trainee along with workout-plan, diet-plan, user-device data, and body measurement for every individual trainee is mentioned in the dashboard, where the authorized users can perform CRUD operation. Similarly, the configuration analysis of blockchain network is also visualized in terms of the number of transactions, blocks, arbitrary nodes, and chain code. Finally, the trainee registration, (such as successful registration and registration request) in the fitness management system monthly-wise is also visualized in the dashboard. This data analytic module provides useful knowledge from the fitness data, which helps in gaining significant market in the fitness industry.

### 7.1. Security Analysis

The security is analyzed against the attacks in the proposed fitness framework.

- Key Attack: the secure fitness framework uses the encryption based on elliptic curve that is used to create the key pair which is difficult to compute by the attacker. The private key generation by solving elliptic curve mechanism requires high computation power, which is difficult for the intruder. The private key is normally distributed among every node for each session agreement.
- False Data Injection Attack: in the proposed blockchain framework, the consensus mechanism is carried out before record validation. Every node verifies and authenticates the integrity of fitness record after successful consensus mechanism.
- Man in Middle Attack: the fitness framework assures and safeguards bilateral authentication and authorization between nodes, as a temporary private key is used for every session agreement, which avoids man in the middle attack.
- Replay Attack: in proposed fitness framework, a separate private key is used for session agreement among nodes. The separate private key prevents the replay attack.

The developed framework provides an intriguing solution for IoT fitness devices. Moreover, the secure framework encompasses data protection, locking access to fitness devices and the consensus mechanism (PBFT), which enables fault tolerance for IoT fitness devices. Furthermore, the data encryption and decentralization functionality of blockchain provides the data security for IoT devices. The smart contracts functionality intensifies trust in IoT fitness devices and scales down the potential costs. Asymmetric encryption is considered to be the underlying technology used to safeguard the blockchain security. The encryption based on asymmetric comprised of public and private key is used to provide the functionality of digital signatures and data encryption. The asymmetric cryptography not only provides a transaction verification and signature, but also ensures the IoT fitness data security in blockchain. The main function of blockchain is to record the data into the block in a secure way where each transaction is verified by the other nodes within the blockchain network.

### 7.2. Performance Evaluation

The working of the proposed intelligent blockchain fitness service is assessed using Hyperledger Caliper [58]. Hyperledger Caliper is a Linux based open-source benchmarking tool that is used to evaluate the performance of the blockchain-based platform. In the designed system, the performance is measured in terms of transaction latency, Transactions Per Second (TPS), and resource utilization. Table 11 summarizes the Hyperledger calliper environment with the technologies used along with the description.

### 7.3. Simulation Results

**Figure 12:**
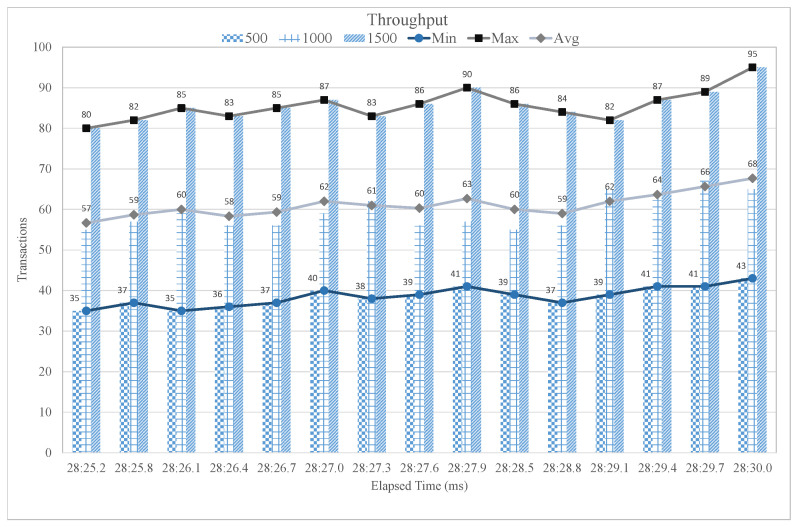
Transaction Per Second.

Figure 12 contemplates the transaction per second (TPS) results, also known as throughput of the proposed blockchain-based intelligent fitness service. The experiments are carried using Hyperledger Caliper, where we have considered different user groups to evaluate the performance of proposed intelligent blockchain-based fitness service. The defined user group is divided into three different sub-groups i.e., 500, 1000, and 1500 users. We also calculated some statistical measure, such as minimum, maximum, and average throughput in order to evaluate the performance of the proposed case study using IoT blockchain platform. Initially, we considered 500 users to evaluate the throughput of the developed blockchain platform. It is observed that the throughput in the case of 500 users group remains stable with a minimum of 35 TPS and maximum of 43 TPS. Similarly, in the case of 1000 users group, the number of transactions are balanced at every elapsed time with a minimum of 57 TPS and a maximum of 68 TPS. Finally, for the 1500 users group, the throughput is also steady with a minimum of 80 TPS and a maximum of 95 TPS. It is observed that the throughput increases as the number of users in the network increases. Moreover, increasing the number of users will not affect the throughput or degrade the system performance in terms of transactions per second.

**Figure 13:**
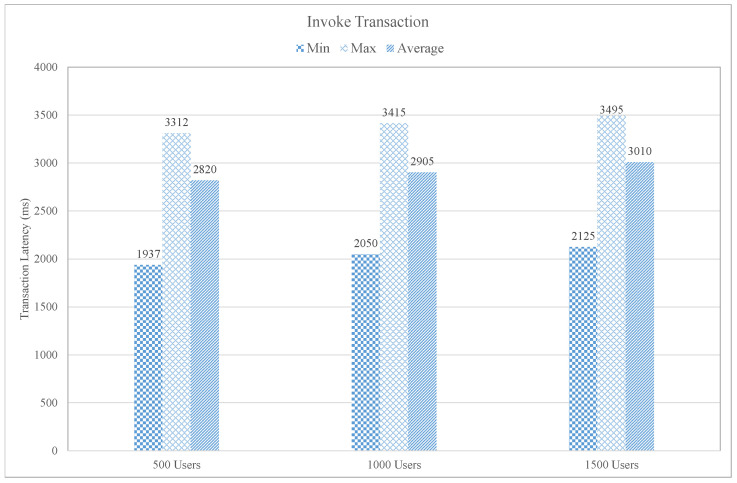
Invoke Transaction Latency.

In Figure 13, the invoked transaction latency of the proposed system concerning three distinct users group is summarized in terms of minimum, maximum, and average latency. The minimum transaction latency in the case of 500 users is 1937 ms, 2050 ms for 1000 users, and 2125 ms for 1500 users. The maximum invoked latency is the latency of the particular request that consumes maximum time to send a request to the blockchain from the client. The maximum latency for different user groups, such as, for 500 users, the maximum latency is 3312 ms, 3415 ms for 1000 users, and 3495 ms for 1500 ms. It is investigated from the graph that the maximum latency slightly increases as the number of users increased. Moreover, the stability and slight increase of latency are due to the intelligent smart contract based on the inference engine that always executes the specific transaction as compared to entire network transactions that improve the proposed blockchain performance.

**Figure 14:**
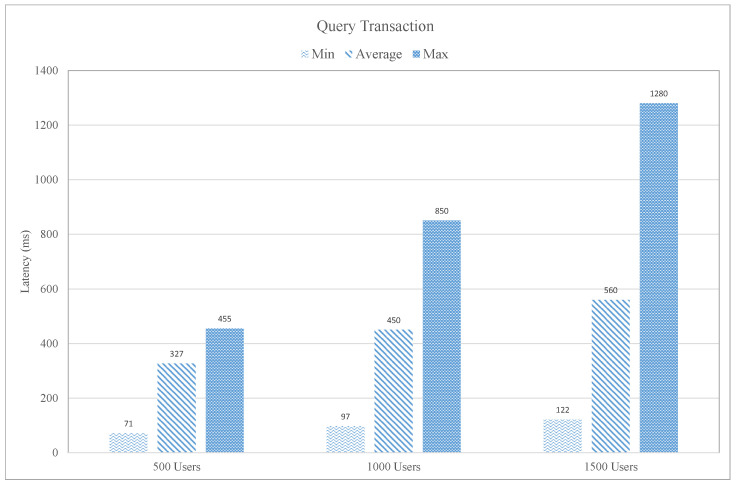
Query Transaction Latency.

In Figure 14, the query function of the proposed intelligent blockchain-based fitness case study is evaluated in terms of latency. We have considered three different users groups to access the performance of the designed blockchain platform. The user-group is 500 users, 1000 users, and 1500 users. The query transaction latency is the time of getting a response from the blockchain network. The query transaction latency for 500 users group in term of minimum, average, and maximum latency is 71 ms, 327 ms, and 445 ms, respectively. Similarly, in the case of 1000 and 1500 user groups, the minimum, average, and maximum latency are 97 ms, 450 ms 850 ms and 122 ms, 560 ms, and 1280 ms, respectively. It is observed from the graph that the average query transaction latency is increased with the increase in the number of user of the blockchain network.

**Figure 15:**
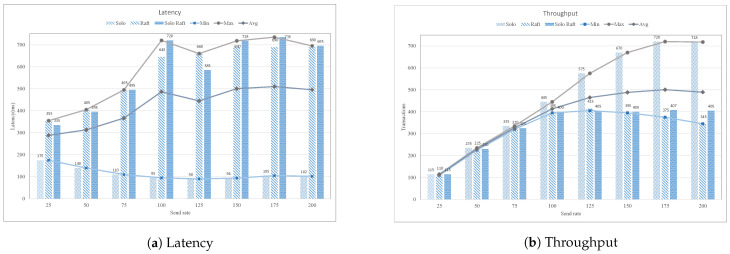
Impact of varying orderer node with different send rate.

Hyperledger Fabric provides the ordering services that are responsible for the ordering of transaction with the help of ordering nodes. The efficiency and performance of the proposed case study are evaluated using three types of ordering service, i.e., solo-raft, solo, and raft with different transaction send rate ranges from 25 TPS–200 TPS, as shown in Figure 15. It is evident from the graph that the latency of raft and solo ordering is higher than the simple solo ordering; this is due to appended transport layer security (TLS), which provides extra security and authentication among peer nodes.

Figure 15a shows the minimum, maximum, and average latency with different transaction send rate. Similarly, Figure 15b presents the throughput of solo, raft, and solo-raft in terms of minimum, maximum, and average throughput. It is found from the graph that the solo order achieves the higher throughput among other ordering services, because it has single node that does not require an additional TLS mechanism.

**Figure 16:**
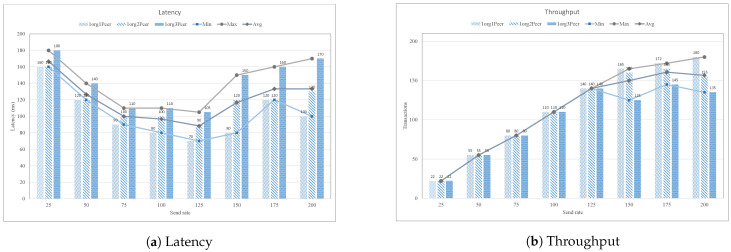
Impact of varying peer node with different transaction rate.

Similarly, in the proposed system, we also carried out several experiments by varying the number of endorser peer in the blockchain network. The endorser peer is a peer node that is responsible for endorsing the proposed transaction. Every transaction before committing is endorsed using the endorser peer function, which is executed by invoking the chaincode. In Figure 16, we evaluated the performance of the proposed case study by varying the peer nodes numbers in terms of average latency and throughput. It is investigated from the graph that the latency of the network is increased as we increase the number of peer nodes in the network. The peer node in the network degrades the network performance in terms of traffic volume, which results in a decline in network throughput. Figure 16a presents the impact of change peer node on the latency of the network between the transaction send rate of 25–200 TPS. It is observed that the increase in latency is directly proportional to the number of peers in the network. If the number of peer’s nodes increases, the traffic volume in the network will also increase, which results in the decline of throughput, as summarized in Figure 16b.

The performance of the proposed case study is also accessed in terms of resource utilization, such as CPU usage (avg,max), memory consumption (avg,max), and traffic in and Traffic, as summarized in Table 12. From the table, it is observed that the proposed blockchain-based intelligent fitness service is working with inadequate resources and improves the network performance by efficiently utilizing system resources.

## 8. Significance and Comparison

The performance evaluation presented in Table 10 shows that the SVM classification model performed well when compared to the other classification model. In order to evaluate the effectiveness of the proposed fitness framework, we further compare the proposed model with other state-of-art approaches discussed in the literature review section. We have consider [35,47,52,54] for comparison with the proposed model in terms of accuracy. Table 13 presents the comparative analysis of the proposed fitness framework with existing models.

## 9. Conclusions and Future Direction

The current functionalities of IoT devices are not efficient enough to defend themselves against threats. This is because of the issues that are involved with resources in IoT devices, immature standards, a lack of secure hardware, and software designs. This work proposes an enhanced smart contract intelligent fitness service in blockchain networks. We developed a secure fitness service using the smart contract enabled blockchain approach to store user personalized fitness data and recommend fitness plans in order to assess the effectiveness of the proposed IoT blockchain platform, such as diet and workout to fitness user. The enhanced smart contract enabled inference engine is used to derive hidden knowledge, such as fat-free mass, body mass index, body fat mass, waist/hip ratio, body fat percentage, and basal metabolic rate based on user and IoT fitness data. The relationship engine is used to compute user’s and IoT fitness device usage data. The developed system used permissioned blockchain network, which solves the inherent issues, which include data scalability, security, and identity, to name a few. Furthermore, an interactive front-end application is developed to expose the fitness services to the client. Finally, for evaluation, we have used Hyperledger calliper, which is used to calculate the performance of the designed platform in terms of throughput, latency, resource utilization, and varying network parameters. Based on experiment, we found that the smart contract integrated inference engine significantly enhances system performance in terms of throughput and resource utilization. This work can be extended in several domains, from healthcare to smart industries. The potential future direction of this work is to evaluate the interoperability of the designed service model with other IoT frameworks. Furthermore, different data storage and consensus algorithms can be considered to observe the data query efficiency and processing rate of the transaction.

## Figures and Tables

**Table 1:** Comparison of Blockchain complexity based on the consensus algorithms.

Consensus Type	Consensus Algorithm	Node Management	Mining Based on	Energy Consumption	Transaction Fee	Validation Speed (s)	Transaction per Second	Applications
Voting-based consensus Algorithm	Raft [8]	Private Blockchain	Random timer	Yes	No	0 s to 10 s	10,000 tps	Smart Contracts
	PBFT [9,10]	Private Blockchain	Mathematical process	Yes	No	0 s to 10 s	2000 tps	Smart Contracts
Proof-based consensus algorithm	PoL [11]	Consortium Blockchain	Prioritized	Yes	Yes	15 s	1000 tps	Crypto-currency
	PoI [12]	Consortium Blockchain	Random value	Yes	Yes	30 s to 1 min	500 tps	IoT application
	PoA [13]	Public Blockchain	Hashing	Partial	Yes	30 s	800 tps	Crypto-currency
	PoS [14,15]	Public Blockchain	Staked owned	Partial	Yes	100 s	1000 tps	Smart contracts, Crypto-currency
	DPoS [12]	Public Blockchain	Staked owned	Partial	Yes	100 s	1000 tps	Bit-shares Crypto-currency
	PoW [16]	Public Blockchain	Hashing	No	Yes	100 s	100 tps	Smart contracts, Crypto-currency

**Table 4:** Body mass index range.

Type	Range
Under weight	*BMI* < 18.5
Normal weight	18.5 ≤ *BMI* ≤ 24.9
Over weight	25 ≤ *BMI* ≤ 29.9
Obesity	30 ≤ *BMI* ≤ 35

**Table 5:** Body fat percentage range.

Type	Gender	Range
Athletes	Male	6% ≤ *BFP* ≤ 13%
	Female	14% ≤ *BFP* ≤ 20%
Average	Male	18% ≤ *BFP* ≤ 24%
	Female	25% ≤ *BFP* ≤ 31%
Obese	Male	*BFP* > 25%
	Female	*BFP* > 32%

**Table 6:** Development environment for the intelligent fitness service based on enhanced smart contract enabled relationship and inference engine in IoT network.

Module	Component	Description
**Intelligent Fitness service**	CPU	Intel(R) Core(TM) i5-8500 CPU @3.00 CHz
	Operating System	Ubuntu Linux 18.04 LTS
	Docker Engine	Version 18.06.1-ce
	Docker-Composer	Version 1.13.0
	IDE	Composer Playground
	Programming Language	Node.js
	Hyperledger Fabric	Version 1.2
	Node Version	8.11.4
	Database	Couch DB
	Memory	12 GB
**Fitness IoT Server**	Hardware	Arduino Uno
	Server	CoAP Server
	Library/Framework	Californium CoAP, Http URL Connection
	Programming Language	Arduino
	Operating System	Ubuntu Linux 18.04 LTS
**IoT Gateway**	Hardware	Raspberry Pi-4
	Server	CoAP Server
	Library/Framework	Californium CoAP, Http URL Connection
**Fitness Blockchain Web Application**	Operating System	Window 10
	Browser	Chrome, Firefox, IE
	Programming Language	HTML, CSS, JavaScript, Node.js
	Library/Framework	Notify.js, Californium CoAP, JQuery, Bootstrap
**Predictive Analytics Model**	Operating System	Microsoft Windows 10
	CPU	Intel(R) Core(TM) i5-8500 CPU @3.00 CHz
	Main Memory	16GB RAM
	Core Programming Language	Python
	IDE	PyCharm Professional 2020
	ML Algorithm	1. Deep Neural Network 2. Support Vector Regressor

**Table 7:** Enhanced smart contract modeling for proposed intelligent fitness service.

Type	Component	Description
**Transaction**	Update Exercise Type	Update the past visit workout plan in record (exercise type, reps)
	Update workout Plan Rep	Update the past visit workout plan in record (exercise type, reps)
	Sensor Reading	Acquired data from IoT fitness devices
	Fat Free Mass	Compute the Fat-Free Mass based on body measurement
	Body Fat Percentage	Compute the Body Fat Percentage based on body measurement
	Body Mass Index	Compute the Body Mass Index based on body measurement
	Waist Hip Ratio	Compute the Waist Hip Ratio based on body measurement
	Basal Metabolism Rate	Compute the Basal Metabolism Rate based on body measurement
	Update Diet Information	Update the Diet information array in record (date, diet products, time)
	Share Record With Trainer	Set the record access permission to a specific Trainer
	Share Record With Trainee	Set the record access permission to a specific Trainee
	Historical Inferred knowledge	Compute the inferred knowledge based on historical data(device and user profile)
**Assets**	Features	Historical inferred knowledge deduce form historical data.
	workout plan	Record of weekly workout plan of trainee
	Device	Iot fitness devices used for acquiring values
	Diet Plan	Record of weekly diet plan of trainee
	Goal	Record of the goal computed based on reading acquired from IoT fitness devices.
	Body Measurement	Record of the body measurement values taken from IoT fitness devices
	Fitness Record	Record the details of the personalized fitness record of trainee along with assigned trainer.
**Participants**	Trainer	Update the Personalized fitness record. Pay membership bills
	Trainee	Update the Personalized fitness record. Create, update the fitness record(fitness devices, diet plan, and workout plan).
	Admin	Create, update the trainee appointment. Create, update the membership bills. Create, update the fitness records. Send the membership bills to trainee.

**Table 8:** RESTFul application programming interface (API) for proposed fitness blockchain platform.

Action	Verb	Media-Type	URI
Fitness Device Management	ALL	Application/JSON	/api/Devices
Fitness Record Management	ALL		/api/fitnessRecord
Diet Plan Management	ALL		/api/DietPlan
Workout Plan Management	ALL		/api/workoutPlan
Body Measurement	GET		/api/bodyMeasurement
Fat Free Mass	GET		/api/FFM
Body Mass Index	GET		/api/BMI
Waist Hip Ratio	GET		/api/WHR/
Basal Metabolic Rate	GET		/api/BMR
Fitness Device Reading	GET, POST		/api/FitnessReading
Historian Record	GET		/api/system/historian
Fetch All Identities	GET		/api/system/identities
Issue Identity to Participant	POST		/api/system/identities/issue
Blockchain Network Test	GET		/api/system/ping

**Table 9:** Summary of the use case for different workout and diet plan recommendation based on the body composition function values.

Case ID	Trainee ID #	Body Composition Function Reading					Recommendation	
		*FFM*	*BFP*	*BMI*	*BMR*	*WHR*	Diet Plan	Workout Plan
1	Trainee 1	26	28	32	1842	1.9	Diet Plan 2	Workout Plan 2
2	Trainee 2	21	23	22	1648	0.97	Diet Plan 1	Workout Plan 1
3	Trainee 3	11	10	20	1552	0.98	Diet Plan 3	Workout Plan 3
4	Trainee 4	30	29	33	1792	1.5	Diet Plan 2	Workout Plan 2
5	Trainee 5	29	27	35	1997	1.2	Diet Plan 2	Workout Plan 2

**Table 10:** Performance evaluation based on reduced set of features using different classification models.

Classifiers (%)	Accuracy (%)	Precision (%)	Recall (%)	F-Measure (%)
DT	85.4	84.2	81.6	82.8
LR	89.3	83.1	84.3	85.3
SVM	92.1	86.5	86.2	87.2
K-NN	84.2	84.0	80.4	82.2

**Table 11:** Environmental Setup of Hyperledger Caliper.

Component	Description
Docker Engine	Version 18.06-ce
CLI Tool	Node-gyp
Docker-Composer	Version 1.130
Node	v8.11.4

**Table 12:** Resource Utilization Analysis of Proposed System.

Type	Name	CPU	CPU	Memory	Memory	Traffic	Traffic
		(max%)	(avg%)	(max)	(avg)	In	Out
Process	local-client.js	11.75	5.25	74.2 MB	72.0 MB	472 KB	142.3 KB
Docker	peer1.Trainer.com	11.92	5.58	79.5 MB	76.7 MB	465.4 KB	139.9 KB
Docker	peer0.Trainee.com	10.65	6.53	412.4 MB	411.4 MB	1.7 MB	923.4 KB
Docker	peer0.Trainer.com	9.88	6.08	411.2 MB	410.1 MB	1.7 MB	919.6 KB
Docker	peer1.Trainee.com	0.00	0.00	8.6 MB	8.6 MB	0 B	0 B
Docker	orderer.com	4.52	1.39	23.5 MB	20.9 MB	1.2 MB	2.3 MB
Docker	ca_nodeGym	0.00	0.00	10.0 MB	10.0 MB	546 B	0 B

**Table 13:** Comparative analysis of the proposed fitness framework with existing models.

Model	Accuracy
[47]	89.5%
[52]	90.45%
[54]	90.3%
[35]	88.45%
**Proposed Fitness Framework**	**92.1%**

## References

[1] Frank L., Engelke P., Schmid T. (2003). Health and Community Design: The Impact of the Built Environment on Physical Activity.

[2] Belza B., Walwick J., Schwartz S., LoGerfo J., Shiu-Thornton S., Taylor M. (2004). pEER REvIEWED: Older Adult perspectives on physical Activity and Exercise: Voices From Multiple cultures. Prev. Chronic Dis..

[3] Bouchard C., Blair S.N., Haskell W.L. (2012). Physical Activity and Health.

[4] Kruk J. (2007). Physical activity in the prevention of the most frequent chronic diseases: An analysis of the recent evidence. Asian Pac. J. Cancer Prev..

[5] Kelly S.J., Ismail M. (2015). Stress and type 2 diabetes: A review of how stress contributes to the development of type 2 diabetes. Annu. Rev. Public Health.

[6] Gholap N., Davies M., Patel K., Sattar N., Khunti K. (2011). Type 2 diabetes and cardiovascular disease in South Asians. Prim. Care Diabetes.

[7] Saghiri A.M., Vahdati M., Gholizadeh K., Meybodi M.R., Dehghan M., Rashidi H. A framework for cognitive Internet of Things based on blockchain. Proceedings of the 2018 4th International Conference on Web Research (ICWR).

[8] Ongaro D., Ousterhout J. In search of an understandable consensus algorithm. Proceedings of the 2014 {USENIX} Annual Technical Conference ({USENIX}{ATC} 14).

[9] Castro M., Liskov B. (1999). Practical Byzantine Fault Tolerance.

[10] Vukolić M. (2015). The quest for scalable blockchain fabric: Proof-of-work vs. BFT replication. International Workshop on Open Problems in Network Security.

[11] Milutinovic M., He W., Wu H., Kanwal M. Proof of luck: An efficient blockchain consensus protocol. Proceedings of the 1st Workshop on System Software for Trusted Execution.

[12] Alsunaidi S.J., Alhaidari F.A. A survey of consensus algorithms for blockchain technology. Proceedings of the 2019 International Conference on Computer and Information Sciences (ICCIS).

[13] Mizrahi I., Rosenfeld M. (2014). Proof of Activity: Extending Bitcoin’s Proof of Work via Proof of Stake. IACR Cryptol. Eprint Arch..

[14] Bartoletti M., Lande S., Podda A.S. A proof-of-stake protocol for consensus on bitcoin subchains. Proceedings of the International Conference on Financial Cryptography and Data Security.

[15] Courtois N.T. (2014). On the longest chain rule and programmed self-destruction of crypto currencies. arXiv.

[16] Nakamoto S. (2008). Bitcoin: A Peer-to-Peer Electronic Cash System. https://bitcoin.org/bitcoin.pdf.

[17] Tang S., Shelden D.R., Eastman C.M., Pishdad-Bozorgi P., Gao X. (2019). A review of building information modeling (BIM) and the internet of things (IoT) devices integration: Present status and future trends. Autom. Constr..

[18] Alam T. (2019). Blockchain and its Role in the Internet of Things (IoT). arXiv.

[19] Lee I., Lee K. (2015). The Internet of Things (IoT): Applications, investments, and challenges for enterprises. Bus. Horizons.

[20] Jamil F., Hang L., Kim K., Kim D. (2019). A novel medical blockchain model for drug supply chain integrity management in a smart hospital. Electronics.

[21] Jamil F., Ahmad S., Iqbal N., Kim D.H. (2020). Towards a Remote Monitoring of Patient Vital Signs Based on IoT-Based Blockchain Integrity Management Platforms in Smart Hospitals. Sensors.

[22] Vermesan O., Friess P. (2013). Internet of Things: Converging Technologies for Smart Environments and Integrated Ecosystems.

[23] West D.M. (2016). How 5G technology enables the health internet of things. Brookings Cent. Technol. Innov..

[24] Zhang Y., Sun L., Song H., Cao X. (2014). Ubiquitous WSN for healthcare: Recent advances and future prospects. IEEE Internet Things J..

[25] Hussain F. (2017). Internet of everything. Internet of Things.

[26] Jamil F., Iqbal M.A., Amin R., Kim D. (2019). Adaptive thermal-aware routing protocol for wireless body area network. Electronics.

[27] Jamil F., Kim D.H. (2019). Improving Accuracy of the Alpha–Beta Filter Algorithm Using an ANN-Based Learning Mechanism in Indoor Navigation System. Sensors.

[28] Ahmad S., Jamil F., Khudoyberdiev A., Kim D. (2020). Accident risk prediction and avoidance in intelligent semi-autonomous vehicles based on road safety data and driver biological behaviours. J. Intell. Fuzzy Syst..

[29] Kranz M. (2016). Building the Internet of Things: Implement New Business Models, Disrupt Competitors, Transform Your Industry.

[30] Jamil F., Iqbal N., Imran, Ahmad S., Kim D. (2021). Peer-to-Peer Energy Trading Mechanism based on Blockchain and Machine Learning for Sustainable Electrical Power Supply in Smart Grid. IEEE Access.

[31] Ahmad S., Jamil F., Iqbal N., Kim D. (2020). Optimal Route Recommendation for Waste Carrier Vehicles for Efficient Waste Collection: A Step Forward Towards Sustainable Cities. IEEE Access.

[32] Iqbal N., Jamil F., Ahmad S., Kim D. (2020). Toward Effective Planning and Management Using Predictive Analytics Based on Rental Book Data of Academic Libraries. IEEE Access.

[33] Chung C.M., Chen C.C., Shih W.P., Lin T.E., Yeh R.J., Wang I. Automated machine learning for Internet of Things. Proceedings of the 2017 IEEE International Conference on Consumer Electronics-Taiwan (ICCE-TW).

[34] Rathore S., Pan Y., Park J.H. (2019). BlockDeepNet a Blockchain-based secure deep learning for IoT network. Sustainability.

[35] Atlam H.F., Walters R.J., Wills G.B. Intelligence of things: Opportunities challenges. Proceedings of the 2018 3rd Cloudification of the Internet of Things (CIoT).

[36] Lee S.W., Prenzel O., Bien Z. (2013). Applying human learning principles to user-centered IoT systems. Computer.

[37] Khan P.W., Byun Y. (2020). A Blockchain-Based Secure Image Encryption Scheme for the Industrial Internet of Things. Entropy.

[38] Griggs K.N., Ossipova O., Kohlios C.P., Baccarini A.N., Howson E.A., Hayajneh T. (2018). Healthcare blockchain system using smart contracts for secure automated remote patient monitoring. J. Med. Syst..

[39] Jamil F., Kim D. (2019). Payment Mechanism for Electronic Charging using Blockchain in Smart Vehicle. Korea.

[40] Jamil F., Iqbal N., Ahmad S., Kim D.H. (2020). Toward accurate position estimation using learning to prediction algorithm in indoor navigation. Sensors.

[41] Xu G., Liu Y., Khan P.W. (2019). Improvement of the DPoS Consensus Mechanism in Blockchain Based on Vague Sets. IEEE Trans. Ind. Informatics.

[42] Khan P.W., Byun Y.C. (2020). Smart Contract Centric Inference Engine For Intelligent Electric Vehicle Transportation System. Sensors.

[43] Wright K.L., Martinez M., Chadha U., Krishnamachari B. SmartEdge: A smart contract for edge computing. Proceedings of the 2018 IEEE International Conference on Internet of Things (iThings) and IEEE Green Computing and Communications (GreenCom) and IEEE Cyber, Physical and Social Computing (CPSCom) and IEEE Smart Data (SmartData).

[44] Salah K., Rehman M.H.U., Nizamuddin N., Al-Fuqaha A. (2019). Blockchain for AI: Review and open research challenges. IEEE Access.

[45] Qian Y., Jiang Y., Chen J., Zhang Y., Song J., Zhou M., Pustišek M. (2018). Towards decentralized IoT security enhancement: A blockchain approach. Comput. Electr. Eng..

[46] Kshetri N. (2017). Can blockchain strengthen the internet of things?. IT Prof..

[47] Rathore S., Kwon B.W., Park J.H. (2019). BlockSecIoTNet: Blockchain-based decentralized security architecture for IoT network. J. Netw. Comput. Appl..

[48] Elliott M., Eck F., Khmelev E., Derlyatka A., Fomenko O. (2019). Physical activity behavior change driven by engagement with an incentive-based app: Evaluating the impact of Sweatcoin. JMIR mHealth uHealth.

[49] Derlyatka A., Fomenko O., Eck F., Khmelev E., Elliott M.T. (2019). Bright spots, physical activity investments that work: Sweatcoin: a steps generated virtual currency for sustained physical activity behaviour change. Br. J. Sport. Med..

[50] Anthony J. (2018). Run2Play. https://www.run2play.com/wp-content/uploads/2018/06/Run2Play_Whitepaper_June-12-2018.pdf.

[51] Holt M. (2017). Movement App: Perfect App for Active Lifestyle. https://icobench.com/ico/movement-app.

[52] Štreit J. (2016). Truegym: Increasing Workout Effectivity. https://truegym.io/wp-content/uploads/2018/08/True-Gym-Whitepaper-EN-v2.pdf.

[53] Blankenship D. (2018). The Hustle App: Promoting Health, Fitness and Wellness. https://www.hustletoken.org/read/HUSL-English-Whitepaper4.1.0e.pdf.

[54] Floyd K. (2018). TeamMate: Gamified Fitness Data. https://icobench.com/ico/teammate.

[55] Seiler B. (2018). Fitrova: Revolutionizing the Health and Fitness Industry. https://icobench.com/ico/fitrova.

[56] Sanchez D. (2019). 180NF: 180º Nutrition and Fitness App. https://icobench.com/ico/180nf.

[57] Maxwell R. (2018). FIT Token: Fitness and Sport Centers. https://icobench.com/ico/fit-token.

[58] Sukhwani H., Wang N., Trivedi K.S., Rindos A. Performance modeling of hyperledger fabric (permissioned blockchain network). Proceedings of the 2018 IEEE 17th International Symposium on Network Computing and Applications (NCA).

